# Vaccination and Vaccinization

**Published:** 1882-02-20

**Authors:** 


### VACCINATION AND VACCINIZATION.

The alarming prevalence of small-pox through certain sections of the country, notwithstanding the very general vaccination which has been practiced during the past few years, will have the effect of stimulating inquiry into the protective influence of the operation as it is ordinarily performed. It is only those who, having declared against the power of vaccinia to protect against variola, and are casting about for arguments to support their position, who will seize upon the existence of the present endemic in the Northwestern States a a prop with which to sustain themselves. Such argument is, however, that of the special pleader, and ill befits the earnest searcher after truth. The fact that a vaccinated person in a given case is seized with small-pox, is not a legitimate argument against vaccination, in the proper meaning of the term. There are many reasons why the operation may have proven a failure:—The virus may not have been of proper quality, and although it may have caused a local sore, that sore may not have had the characteristics of the vaccinia pustule. This, we believe, s a not uncommon occurrence. The effects of vaccinia may have disappeared from the system; they evidently do pass from the systems of some sooner than from others, as attested by the results of re-vaccination. Perhaps humanized virus, which has passed through many systems, has been employed, and, the views of Jenner to the contrary notwithstanding, it seems to be no longer doubtful that humanized virus undergoes deterioration in its passage through the system, and is not as safe a protective as that which has been taken directly from the heifer.

But all of the above conditions may be observed, and precautions taken, and still the vaccinated person not be proof against small-pox. A recent German writer has submitted a plausible suggestion as to the cause of the incomplete immunity in such cases. The person, he says, although vaccinated, is not "vaccinized"—the latter term being that by which he designates such a changing of the system with vaccinia as to overcome the susceptibility to variola. A series of carefully conducted experiments has convinced him that a not inconsiderable proportion of those vaccinated are not vaccinized. He now recommends, and in cases where he has authority, compels successive vaccinations until the susceptibility to the virus has completely disappeared, as indicated by absence of the slightest trace of the essential character of the vaccine pustules at the point of application. He has found that, in some, sores more or less characteristic may be produced until the third vaccination, and holds that as long as such a sore is possible, the person is susceptible to small-pox.

These observations are pregnant with suggestions, and perhaps we have in them the removal of the weighty argument against vaccination, which exists in the fact that vaccinated persons not infrequently die of variola, the vaccination not having vaccinized. —[Michigan Med. News.

